# Kinetics of Phenol Biodegradation by Heavy Metal Tolerant Rhizobacteria *Glutamicibacter nicotianae* MSSRFPD35 From Distillery Effluent Contaminated Soils

**DOI:** 10.3389/fmicb.2020.01573

**Published:** 2020-07-15

**Authors:** Purushothaman Duraisamy, Jegan Sekar, Anu D. Arunkumar, Prabavathy V. Ramalingam

**Affiliations:** Microbiology Lab, Biotechnology Programme, M. S. Swaminathan Research Foundation, Chennai, India

**Keywords:** phenol biodegradation, distillery effluent, *Glutamicibacter* sp., *Canna indica*, Haldane’s kinetics, heavy metal tolerance, soil microcosm

## Abstract

Biodegradation of phenol using bacteria is recognized as an efficient, environmentally friendly and cost-effective approach for reducing phenol pollutants compared to the current conventional physicochemical processes adopted. A potential phenol degrading bacterial strain *Glutamicibacter nicotianae* MSSRFPD35 was isolated and identified from *Canna indica* rhizosphere grown in distillery effluent contaminated sites. It showed high phenol degrading efficiency up to 1117 mg L^–1^ within 60 h by the secretion of catechol 1,2-dioxygenase via ortho intradial pathway. The strain MSSRFPD35 possess both the catechol 1,2 dioxygenase and catechol 2,3 dioxygenase coding genes that drive the *ortho* and *meta* pathways, but the enzymatic assay revealed that the strain cleaves catechol via *ortho* pathway. Haldane’s kinetic method was well fit to exponential growth data and the following kinetic parameter was obtained: μ^∗^ = 0.574 h^–1^, *K*_i_ = 268.1, *K*_s_ = 20.29 mg L^–1^. The true μ_max_ and *S*_m_ were calculated as 0.37 h^–1^ and 73.76 mg L^–1^, respectively. The Haldane’s constant values were similar to earlier studies and healthy fitness depicted in correlation coefficient value *R*^2^ of 0.98. Phenol degrading kinetic’s was predicted using Haldane’s model as *q*_max_ 0.983, *K*_i_′ 517.5 and *K*_s_′ 9.152. Further, MSSRFPD35 was capable of utilizing different monocyclic and polycyclic aromatic hydrocarbons and to degrade phenol in the presence of different heavy metals. This study for the first time reports high phenol degrading efficiency of *G. nicotianae* MSSRFPD35 in the presence of toxic heavy metals. Thus, the strain *G. nicotianae* MSSRFPD35 can be exploited for the bioremediation of phenol and its derivatives polluted environments, co-contaminated with heavy metals.

## Introduction

Phenol and its derivatives namely nitrophenol, halogenated phenol, alkylphenol, etc. are widely used in several industrial plants like petrochemical, phenol resin, pharmaceuticals, paint, textile, leather, pulp mills, coal conversion, and leather processing units (Haddadi and Shavandi, 2013; Jiang et al., 2013; Villegas et al., 2016; Prasse et al., 2018). Gallons of unprocessed effluents polluted with phenol and its derivatives discharged by these industries are reported to contaminate soil, groundwater table and agriculture lands; and to harm the soil and plant health and productivity (Shi et al., 2014; Wu et al., 2018) and also to affect terrestrial and aquatic animals, and humans at very low concentrations (Prasse et al., 2018).

Phenolic compounds have a recalcitrant structure, which consists of an aromatic ring with a hydroxyl group attached to the benzene ring; making it resistant to natural biodegradation and decomposition (Reardon et al., 2000; Ma et al., 2013; Chen et al., 2017). Phenol is highly soluble in water up to the concentration of 10 g L^–1^ (Bajaj et al., 2009) and thus the effluents discharged from industries contain high concentrations of phenol and its derivatives in the range of 50–2000 mg L^–1^ (Jusoh and Razali, 2008) concentrations much higher than the permissible limits leading to high risk of polluting the environment. The permissible limits of phenol in industrial effluents to be discharged in the domestic surface water is only 1 mg L^–1^ (IS: 2490-1974) and 5 mg L^–1^ in public sewers (IS: 3306-1974) (Hussain et al., 2015) and concentration range of 5–2000 mg L^–1^ phenol is reported to be carcinogenic for human and toxic to all life forms (Comte et al., 2013).

In addition to phenol, the effluent released from industries contain heavy metals like copper, lead, cadmium, chromium, etc., as co-contaminants in different composition and concentration which are highly toxic, persistent, and non-degradable in nature (Thavamani et al., 2012; Wong et al., 2015). The continued discharge of phenol and heavy metal contaminated industrial effluents leads to its accumulation in the environment including water bodies that reach a threshold harmful to living systems, and acute phenol exposures causes lung and digestive tract carcinoma, liver, kidney, heart, and nervous system disorders in human, enters the food chain and leads to severe socio-environmental problems (Wang et al., 2011; Fan et al., 2017). Therefore, it is imperative to reduce the concentration of phenol and heavy metals in the industrial effluents and maintain defined standards before releasing these pollutants into the environment.

Although numerous physio-chemical methods such as water chlorination, flocculation, photocatalysis, Fenton’s reaction, ozonisation, chemical oxidation, activated carbon adsorption, reverse osmosis, ion exchange with resin, etc., are widely adopted for the removal of phenol and heavy metal contaminants from industrial waste-waters (Mohammadi et al., 2014; Villegas et al., 2016) these approaches are less efficient with high operation cost and produce intermediate compounds as secondary pollutants (Rajasulochana and Preethy, 2016). Therefore biological treatment especially the use of microbial cultures is reported to significantly degrade phenol and its derivatives from different industrial wastewaters, particularly bacterial embedded treatments showed a significant reduction at low cost without secondary pollutants, and is eco-friendly (Banerjee and Ghoshal, 2010b; Wang et al., 2015; Chen et al., 2017; Tiwari et al., 2017).

Several bacterial groups are described to degrade phenol either by anaerobic or aerobic metabolic activity and utilize it as a sole energy source (Thavamani et al., 2012; Wang et al., 2015; Gu, 2016). Phenol degrading bacteria follow either the *ortho* cleavage pathway which converts catechol into intermediate *cis, cis* muconic acid or the *meta* cleavage pathway that converts catechol to 2-hydroxymuconic semialdehyde (2-HMSA) (Hamzah and Al-Baharna, 1994; Banerjee and Ghoshal, 2010b; Hasan and Jabeen, 2015; Wu et al., 2018) during phenol degradation. Different phenolic compounds at varying concentrations are reported to trigger either *ortho* or *meta* or both the metabolic cleavage pathways involved in phenol degradation (Wu et al., 2018). *Pseudomonas cepacia* ATCC 29351, when grown on salicylate, activates only the *ortho*-pathway, while benzoate activates both *ortho* and *meta* pathways (Hamzah and Al-Baharna, 1994; Mahiudddin et al., 2012). *Pseudomonas putida* ATCC 49451 degrades benzoate at 200–300 mg L^–1^ involving only the *ortho* pathway, but at higher concentrations of benzoate, the degradation involves both the pathways (Cao et al., 2008). Bacterial degradation of phenol and its derivatives like benzene and toluene had been inferred and extensively studied in different groups such as *Pseudomonas* sp. (Reardon et al., 2000; Mahiudddin et al., 2012; Hasan and Jabeen, 2015; Chen et al., 2017; Iqbal et al., 2018), *Burkholderia* sp. (Arora and Jain, 2012), *Kocuria* sp. (Wu et al., 2018), *Acinetobacter* sp. (Jiang et al., 2013; Iqbal et al., 2018), *Arthrobacter* sp. (Wong et al., 2015), *Bacillus* sp. (Banerjee and Ghoshal, 2010b; Hasan and Jabeen, 2015; Iqbal et al., 2018), *Halomonas* sp. (Haddadi and Shavandi, 2013), *Arthrobacter* sp. W1, etc. (Ma et al., 2013; Shi et al., 2014; Wong et al., 2015).

Degradation kinetics of microbial bioremediation in bioreactor offers evidence for optimum design and efficient bioremediation of phenol contaminated effluents (Banerjee and Ghoshal, 2010b). Generally, the derivatives of phenol and other co-contaminants such as heavy metals discharged from industries pose great challenge for efficient biodegradation and also depends on the tolerance level of the degrading bacterial isolates as these toxic substances negatively correlate with the degradation efficacy (Thavamani et al., 2012; Mohammadi et al., 2014; Satchanska et al., 2015; Wong et al., 2015; Chen et al., 2017; Iqbal et al., 2018). However, only few studies have reported microbial degradation of phenol particularly at high concentrations (i.e.) up to 1100 mg L^–1^, and limited studies have reported the biological degradation efficiency of phenol under the influence of different heavy metals such as chromium, etc., till date (Lima et al., 2008; Ontanon et al., 2015; Wong et al., 2015). Moreover, few studies have been carried out to assess the phenol degrading efficiency of bacteria isolated from distillery effluent contaminated *Canna indica* rhizosphere associated soil and to estimate the impact of heavy metals on the rate of phenol degradation. Hence exploration of strains with the capability to degrade phenol with high efficiency and with tolerance to multiple heavy metals can be promising candidates for the bioremediation of phenol contaminated effluents co contaminated with heavy metals which have inhibitory effects on bacterial degradation. So, this study aims (i) to isolate and characterize potential phenol degrading bacterial isolates, (ii) to evaluate kinetic models to determine the degradation efficiency of potential isolates, (iii) to identify molecular and metabolic pathways involved in the degradation of phenol, and (iv) to determine the impact of heavy metals pollutants in phenol biodegradation.

## Materials and Methods

### Materials and Chemicals

Davis minimal medium (DMM), 4-aminoantipyrine, potassium ferrocyanide, catechol, sodium hydroxide pellets used in this study were purchased from HiMedia Laboratories Pvt. Ltd. Mumbai, phenol, 2-mercaptoethanol, ammonium hydroxide, glycerol, disodium EDTA, primers, acids and solvents were procured from Sigma, Aldrich, Tris Base, Agarose from Bio Basic Inc. All the following heavy metals CoCl_2_.2H_2_O, ZnSO_4_.H_2_O, (CH_3_COO)_2_Pb.3H_2_O, HgCl_2_, CdCl_2_, CuSO_4_.5H_2_O, NiCl_2_.6H_2_O, MnCl_2_.4H_2_O, and K_2_Cr_2_O_7_ were purchased from Merck Millipore, Taq DNA polymerase Master Mix from Ampliqon and PCR purification Kit from Favorgen Biotech Corp. were used.

### Isolation of Bacteria From Rhizosphere Soil

Phenol degrading bacterial strains were isolated from rhizosphere soils of *Canna indica* grown in sites contaminated by effluents from Distillery plant located in Vuyyuru, Krishna District, Andhra Pradesh, India (16°21′52.7″N 80°51′52.2″E). Three individual *C. indica* plants were uprooted and the rhizosphere soils from each sample were collected aseptically in sterile polythene bags and pooled. About 10 g of rhizosphere soil was used for the isolation of phenol degrading bacterial isolates by aseptically dispensing it into 100 ml of DMM containing dipotassium phosphate (7 g L^–1^), monopotassium phosphate (2 g L^–1^), ammonium sulfate (1 g L^–1^), sodium citrate (0.5 g L^–1^) and magnesium sulfate (0.1 g L^–1^) with final pH 7.0 supplemented with 200 mg L^–1^ of phenol and enriched by incubating in the shaker at 160 rpm at 30°C for 24 h. Then 1 ml of the enriched soil suspension sample was serially diluted and spread on DMM plate amended with 200 mg L^–1^ of phenol as the sole source of carbon with 1.8% agar and incubated for 48 h at 30°C and the colony-forming units (CFU) were counted. Diverse individual colonies were selected and streaked in 200 mg L^–1^ phenol amended DMM medium and stored in 25% glycerol (v/v) at −80°C for further analysis.

### Screening for Potential Phenol Degrading Strains

The bacteria isolates obtained from the DMM medium amended with 200 mg L^–1^ of phenol were screened to determine the phenol degrading efficiency by inoculating in DMM agar plates amended with different phenol concentration of 600, 700, 800, 900, 1000, and 1200 mg L^–1^. The inoculated plates were incubated at 30°C for 72 h and the isolates with efficiency to grow in phenol concentration of 1200 mg L^–1^ were selected and maintained as pure culture (Sandhu et al., 2009).

### DNA Fingerprinting Analysis

Genomic DNA was isolated from efficient bacterial isolates capable of growing in 1000 mg L^–1^ of phenol and the diversity was analyzed using BOXA1R primer (5′-ACG GCA AGG CGA CGC TGA CG-3′) (Viswanath et al., 2015). Each 20 μl reaction containing 6 μl of 2X ampliqon red mix, 2 μl of 2.5 pmol primer, 2 μl of 50 ng template DNA was made up to 20 μl using double sterilized HPLC water. The PCR was carried by, initial denaturation at 94°C for 5 min, 35 cycles of 94°C for 3 s, 92°C for 30 s, annealing at 50°C for 1 min, extension at 68°C for 8 min, followed by final extension for 10 min then hold at 4°C. Around 20 μl of PCR products were electrophoresed for 6 h on 2% agarose gel prepared in 1X TAE buffer. The BOX-PCR DNA profiles were visualized under UV illumination and documented using a Gel Doc^TM^ XR+ Gel Documentation System (Bio-Rad, United States). The fingerprinting profiles were analyzed using GelJv.2 DNA tool by normalization, recognition, and assignment of bands on the gel by the Dice coefficient (Heras et al., 2015). The cluster analysis was performed by unweighted pair group method with arithmetic mean (UPGMA) algorithm and the dendrogram was constructed with similarity matrices.

### 16S rRNA Based Identification of the Phenol Degrading Bacterial Isolates

The 16S rRNA gene was amplified from the representative isolates of the BOX-PCR cluster groups using universal primers 27F (5′-AGA GTT TGA TCM TGG CTC AG-3′) and 1492R (5′-TAC GGH TAC CTT GTT ACG ACT T-3′) (Sekar et al., 2018). Each 20 μl reaction containing 6 μl of 2X ampliqon red mix, 2 μl of 2.5 pmol 27F and 1492R primers, 2 μl of 50 ng template DNA was made up to 20 μl using double sterilized HPLC water. The PCR conditions were as follows, initial denaturation 94°C for 5 min, 35 cycles of 94°C for 1 min, 55°C for 1 min and 68°C for 8 min. followed by final extension for 10 min then hold at 4°C. PCR products were electrophoresed on 1% agarose gel in 1X TAE buffer at 100 V for 30 min and amplified PCR products were purified using the FavorPrep GEL/PCR Purification Kit (Taiwan) and were sequenced. The taxonomic position of the isolates were identified by sequence similarity blast search against EzTaxon sequence database (Kim et al., 2012). The 16S rRNA phylogenetic tree was constructed using a neighbor-joining algorithm and confidence level in nodes were determined using 1000 bootstrap resampling conducted using the MEGA v6 (Kumar et al., 2016).

### Determination of Growth, Phenol Degradation, and Quantification of Substrate

The growth and phenol degrading efficiency of representative isolates from each cluster groups of BOX profiles were determined in DMM broth with 1000 mg L^–1^ phenol. The culture inoculated broths were incubated in 30°C at 160 rpm for 96 h, the sample were withdrawn at every 24 h and analyzed for phenol concentration. A 1 ml of culture was centrifuged at 8000 rpm for 10 min to remove the cell debris and 50 μl of supernatant was made up to 1 ml and used to determine the phenol residual content through modified 4-amino antipyrine method at absorbance 510 nm by adding 25 μl of 0.5 N NH_4_OH to sample, followed by 15 μl of PBS (pH-6.9), 11 μl of 4-aminoantipyrine, and 11 μl of potassium ferrocyanide and incubated at 30°C room temperature for 15 min. The appearance of dark red color indicated positive reaction and intensity was measured using Multiskan^TM^ GO Microplate Spectrophotometer at 500 nm (APHA, 2017). Phenol standard was prepared with the concentration range of 100 mg L^–1^–1200 mg L^–1^, linear equation *Y* = 0.002447^∗^*X* + 0.1423 was obtained and used to determine phenol concentration of unknown samples (Supplementary Figure S1).

### Assessment of Growth Rate and Phenol Degradation Kinetics of MSSRFPD35

The biodegradation of phenol by a potential isolate MSSRFPD35 was determined in batch mode in 250 ml Erlenmeyer flask containing 95 ml of DMM with initial phenol concentration ranging from 0 to 1117 mg L^–1^. A 5% inoculum of MSSRFPD35 (8 log CFU ml^–1^) grown in DMM broth with phenol (200 mg L^–1^) was inoculated in DMM medium amended with phenol concentrations of 0 to 1117 mg L^–1^, flask without inoculation served as controls and the assay was performed in triplicates. The inoculated flasks were incubated on a rotary shaker at 160 rpm for 120 h at 30°C. At every 12 h time interval samples were withdrawn from each conical flask and the growth rate was measured as absorbance at 600 nm using Multiskan^TM^ GO Microplate Spectrophotometer (APHA, 2017).

### Cell Growth Rate Kinetics

The growth absorbance of MSSRFPD35 was converted into dry biomass using linear coefficient derived from growth absorbance vs. dry biomass (*X*), the cell growth was follows first-order kinetics.

(1) $$\frac{d\,X}{d\,t}=\mu \,X$$

(2) d⁢X/X=μ⁢d⁢t

when integrating Eq. (2) with limit *t*_0_ (initial time) to *t* (a time when maximum biomass reached), it becomes

(3) $$\text{ln}\,X-\text{ln}\,X_{0}=\mu \,\left(t_{0}-t\right)$$

where *X*_0_ – initial biomass, *X* – biomass at time *t*, and Eq. (3) rewritten as

(4) $$\mu =\text{ln}\,\left(X_{0}/X\right)/\Delta \,t$$

The experimental specific growth rate (μ) was calculated from the slope of a semi-logarithmic plot of dry biomass ln(*X*/*X*_0_) vs. time.

The growth rate of microbes on inhibitory substrates such as phenol is often described using the substrate inhibition model especially the Haldane’s kinetic model Eq. (5)

(5) $$\mu =\frac{\mu^{*}\,S}{K_{s}+S+\left(S^{2}/K_{i}\right)}$$

Experimental μ was calculated for each initial phenol concentration and this μ value against different initial phenol concentration (*S*_0_) was used to predict various kinetic parameters of Haldane’s kinetic model using non-linear regression analysis.

Here μ^∗^ is one of the fitting parameters of the Haldane model, the true μ_max_ occurs when *dμ*/*dS* = 0;

(6) $$S_{m}=\sqrt{K_{s}\,K_{i}}$$

Replacing Eq. (6) in Eq. (5), (Christen et al., 2012).

(7) $$\mu_{\max}=\frac{\mu^{*}}{1+2\,\sqrt{K_{s}/K_{i}}}$$

Where μ_max_ is true maximum growth rate, calculated from Eq. (7).

### Degradation Kinetics

The phenol degradation rate from each sample was estimated by using 4-aminoantipyrine method as described above (APHA, 2017). Reduction of phenol content is non-linear concerning time, first-order kinetics was adopted for degradation.

(8) $$\frac{d\,S}{d\,t}=-q\,S$$

(9) d⁢S/S=-q⁢d⁢t

Integrate Eq. (9) with time *t*_0_ to *t* (when complete depletion of phenol)

(10) $$\text{ln}\,S_{0}-\text{ln}\,S=-q\,\left(t_{0}-t\right)$$

where *S*_0_ – initial phenol concentration, *S* – phenol concentration at time *t*.

(11) $$q=\text{ln}\,\left(S/S_{0}\right)/\Delta \,t$$

To determine the degradation rate (*q*) initial phenol concentration (*S*_0_) was acquired from the slope of the linear curve by plotting between −ln(*S*/*S*_0_) and time. The phenol degradation kinetics was acquired by experimental degradation rate of individual phenol concentration (Banerjee and Ghoshal, 2010b; Satchanska et al., 2015). To predict the coefficient values, Haldane’s inhibitory model was used (Eq. 12),

(12) $$q=\frac{q^{*}\,S}{K_{s}^{\prime }+S+S^{2}/K_{i}^{\prime }}$$

Where *K*_s_′ saturation constant, *K*_i_′ Inhibition constant, *S* substrate conc. at time *t.*

### Molecular Metabolic Pathway Identification

The degradative enzymes involved in phenol degradation was assessed by targeting the genes coding for catechol 1,2-dioxygenase (CBT77506) and catechol 2,3-dioxygenase (PJJ43550). The nucleotide sequences of these enzymes from *Glutamicibacter* spp. were extracted from the European Nucleotide Archives (ENA). Primers were designed for catechol 1,2-dioxygenase gene between 321 bp and 730 bp AC12O-F (5′-ATC GAA GGC CCT TAC TAC-3′); AC12O-R (5′-AAG TAC AGC TGG GCG GTG A-3′) and for catechol 2,3-dioxygenase between 3 bp to 819 bp AC23O-F (5′-GAG CAA AGA GAT CGC AAA CC-3′); AC23O-R (5′-GTA GAT CTC GAT GCG GTG GT-3′) using Primer3Plus program (Untergasser et al., 2012). PCR was carried out in a Bio-Rad thermal cycler with 20 μl of reaction mixture containing 2.5 pmol of each forward and reverse primer, ∼50 ng of DNA template, 1X Ampliqon Taq DNA Polymerase Master Mix RED, and the amplification conditions were as follows, an initial denaturation for 5 min at 94°C, followed by 35 cycles at 94°C for 1 min, 56°C for 1 min and 72°C for 1 min, with a final extension at 72°C for 10 min. 5 μl of the PCR product from each sample was electrophoresed using 1% (w/v) agarose gel with 100 bp marker (Thermo, India) and later purified using FavorPrep GEL/PCR Purification Kit (Taiwan). The purified products were sequenced using capillary electrophoresis on an ABI 310 Genetic Analyzer (Applied Biosystems). The identities of the sequenced fragments were determined through BLASTN analysis and CLUSTALW alignment was performed using similarity sequences and the phylogenetic relationship was determined using the neighbor-joining method, with bootstrap analysis (1000 data sets) through Molecular Evolutionary Genetics Analysis (MEGA 6) (Kumar et al., 2016).

### Detection of Enzymatic Cleavage Pathway Involved in Phenol Degradation

The strain MSSRFPD35 was inoculated in DMM medium containing 1000 mg L^–1^ of phenol and harvested at late exponential phase (48 h) by centrifugation at 8000 rpm at 4°C for 10 min. The bacterial cells were washed and resuspended in Tris-HCl buffer (pH 7.6) and kept in ice to avoid heat generation during sonication for 4 min (1 or 2 min off) and to achieve complete lysis. The intracellular crude enzyme present in supernatant were separated from cell debris by centrifugation at 8000 rpm 4°C for 10 min and the cell-free extracts were stored at −20°C. Catechol 1,2-dioxygenase (*Ortho* enzymatic cleavage assay) and catechol 2,3-dioxygenase (*Meta* enzymatic cleavage assay) enzyme activities were spectrophotometrically determined using 3.5 ml of quartz cuvette as described by Feist and Hegeman (1969).

The enzymatic assay for *Ortho* cleavage pathway was performed using 0.7 ml of distilled water, 2 ml of 50 mM Tris-HCL, 0.1 ml of 100 mM β-mercaptoethanol, 0.1 ml of 1 mM catechol and 0.1 ml of cell-free extract. The formation of aromatic ring cleavage product *cis, cis* muconic acid was measured spectrophotometrically at an absorbance of 260 nm. Similarly, the enzymatic assay for *meta* cleavage pathway was carried out using 0.6 ml of distilled water, 2 ml of 50 mM Tris-HCl, 0.2 ml of 100 mM catechol and 0.2 ml of cell-free extract mixed well and the increase in absorbance was measured at 375 nm for 5 min. The formation of 2-HMSA from catechol indicates the presence of catechol 2,3-dioxygenase activity at an absorbance of 375 nm. Enzyme activity and specific activities were calculated by equation described by Bhardwaj et al. (2015).

### Heavy Metal Tolerance Assay

Heavy metal tolerance and phenol degradation potential of MSSRFPD35 were determined using manganese (II) MnCl_2_.4H_2_O – 800 mg L^–1^; iron (II) FeSO_4_ – 400 mg L^–1^; Zinc (II) ZnSO_4_.H_2_O – 600 mg L^–1^; lead (II) (CH_3_COO)_2_ Pb.3H_2_O – 200 mg L^–1^; cadmium (II) CdCl_2_ – 50 mg L^–1^; chromium (VI) K_2_Cr_2_O_7_ – 50 mg L^–1^; nickel (II) NiCl_2_.6H_2_O – 20 mg L^–1^; copper (II) CuSO_4_.5H_2_O – 20 mg L^–1^; cobalt (II) CoCl_2_.2H_2_O – 10 mg L^–1^; and mercury (II) HgCl_2_ – 10 mg L^–1^ in DMM medium with 1000 mg L^–1^ of phenol. The flasks were inoculated with 5% inoculum of *G. nicotianae* MSSRFPD35 (8 log CFU ml^–1^) and incubated on a rotary shaker at 160 rpm for 72 h at 30°C. DMM medium without heavy metals and inoculation were maintained as control and all the treatments were performed in triplicates. The samples from flasks were collected at every 24 h intervals and phenol concentration was measured.

### Growth on Different Aromatic Substrates

Growth of MSSRFPD35 in different phenol derivatives like tannic acid 50 mg L^–1^; cinnamic acid 100 mg L^–1^; 1-chloro-2,4-dinitro benzene 50 mg L^–1^; 4-nitrophenol 50 mg L^–1^; catechol 100 mg L^–1^; 1-naphthol 50 mg L^–1^; naphthylamine 50 mg L^–1^; and gallic acid 200 mg L^–1^ was screened by inoculating 5% inoculum (8 log CFU/ml) of MSSRFPD35 in DMM broth containing phenol derivatives at the above-mentioned concentration and incubated at 30°C on a rotary shaker at 160 rpm. Bacterial growth was measured at 600 nm absorbance (Multiskan^TM^ GO, Thermo Scientific) after 72 h of incubation. Individual derivatives in DMM without inoculum served as negative control and were performed in triplicates.

### Microcosm Study

Microcosms were set up by adding 50 g of soil in 250 ml conical flask and sterilized with three cycles of autoclaving. A uniform volume of 17.5 ml at different concentrations (65 mg L^–1^, 110 mg L^–1^ and 240 mg L^–1^) of phenol in sterile distilled water and 2.5 ml inoculum with 8 log_10_ CFU ml^–1^ of *G. nicotianae* MSSRFPD35 were added into the respective flask under aseptic condition and mixed thoroughly to get a uniform suspension of soil, phenol, and the inoculum. The treatments without microbial inoculum served as control and were performed in triplicates. The flasks were kept in 30°C for 10 days with gentle mixing at every 24 h. About 500 mg of soil samples were collected at the time of inoculation and on the 10th day of incubation from each treatment and suspended in 500 μl of distilled water, vigorously mixed and phenol was extracted by collecting the aqueous phase of the mixture and the phenol concentration was determined from the extracted samples as described above (APHA, 2017).

### Statistical Analysis

All experiments were performed in triplicates, regression analysis and kinetics methodology were done using GraphPad Prism 6. The analytical results were compared by applying a one-way ANOVA and significant differences among treatments were determined by Student’s *t*-test.

### Strains and Gene Sequences Submission

The isolated strains from this study were submitted to M. S. Swaminathan Research Foundation Culture Collection [WDCM Registered Number (1220) and accession are provided, MSSRFPD35 = MSSRFCC1542]. The gene sequences from this study were submitted to GenBank/EMBL and the accession numbers for the 16S rRNA gene of the phenol degrading bacterial are KY849351, KY849352, KX901882 – KX901885, catechol 1,2-dioxygenase gene – MK656957 and catechol 2,3-dioxygenase gene – MK656958.

## Results

### Isolation and DNA Fingerprinting of Phenol Degrading Bacteria

The enriched *Canna indica* rhizosphere soil suspension spread plated on DMM agar medium amended with 200 mg L^–1^ of phenol yielded 7.3 log_10_ CFU ml^–1^ after 48 h of incubation at 30°C. Around 128 different morphotype bacterial colonies were isolated and the phenol tolerance assay revealed that 11 isolates which were the most dominant colonies could grow in 1000 mg L^–1^ of phenol concentration and were coded as MSSRFPD27, MSSRFPD28, MSSRFPD29, MSSRFPD30, MSSRFPD35, MSSRFPD36, MSSRFPH100, MSSRFPH124, MSSRFPH134, MSSRFPH139, and MSSRFPH145. The BOX-PCR fingerprinting analysis showed the existence of polymorphism by amplifying three distinct patterns among the isolates. Dendrogram generated based on BOX-PCR profile with Dice coefficient and the UPGMA clustering method with 90% tolerance level revealed the existence of three diverse cluster groups (Cluster I, II, and III) at 85% similarity coefficient (Supplementary Figure S2).

### Taxonomical Identification

**FIGURE 1:**
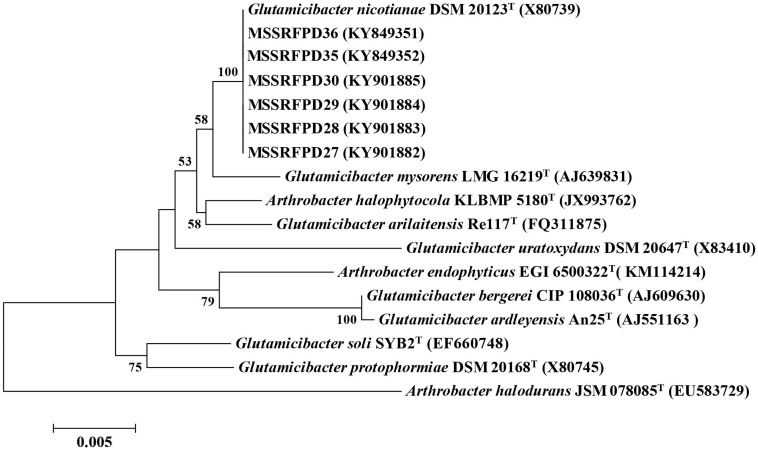
Neighbor-joining tree based on partial 16S rRNA gene sequences of phenol degrading strains and other closely related strains.

Two isolates from each BOX-PCR cluster groups were chosen and the 16S rRNA was amplified and sequenced for taxonomical identification. The EzTaxon analysis revealed that representative isolates of cluster groups I (MSSRFPD27 and MSSRFPD30), II (MSSRFPD28 and MSSRFPD36), and III (MSSRFPD29 and MSSRFPD35), showed 99.9% identity to *G. nicotianae* DSM 20123^*T*^, followed by 99.45% similarity index with *G. mysorens* LMG 16219^*T*^ and 99.24% with *G. arilaitensis* Re117^*T*^. Phylogenetic analysis of 16S rRNA of the phenol degrading isolates and closest type strains sequence revealed the isolates from cluster groups I, II, and III formed a monophyletic clade with *G. nicotianae* DSM 20123^*T*^ which indicates that MSSRFPD36 and other isolates belong to the group of *G. nicotianae* with high phenol degrading efficiency (Figure 1).

### Biodegradation of Phenol

**FIGURE 2:**
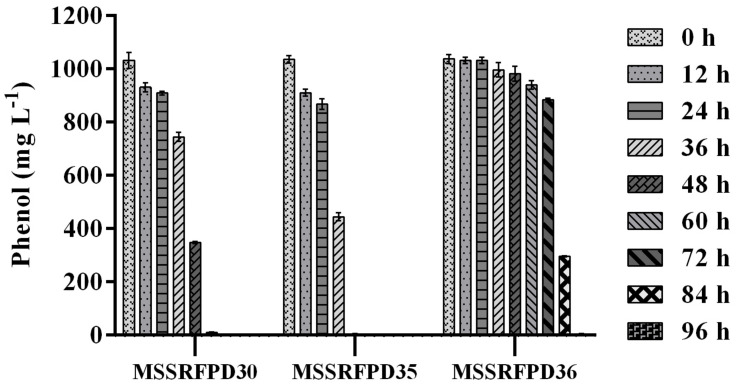
Determination of phenol degradation efficiency of representative isolates from each BOX PCR cluster groups at 1000 mg L^–1^ phenol.

Based on the BOX-PCR and 16S rRNA analysis 3 polymorphic isolates MSSRFPD30, MSSRFPD35, and MSSRFPD36 were used to determine the phenol degrading efficiency and rate of degradation at 1000 mg L^–1^. Among the three strains, MSSRFPD35 rapidly degrade the 1000 mg L^–1^ of phenol completely within 48 h of incubation, followed by MSSRFPD30 and MSSRFPD36 in 60 h and 96 h, respectively (Figure 2). This indicated strain MSSRFPD35 is highly potential and an efficient phenol degrading isolate obtained from this study.

### Growth Kinetics and Degradation Kinetics

**FIGURE 3:**
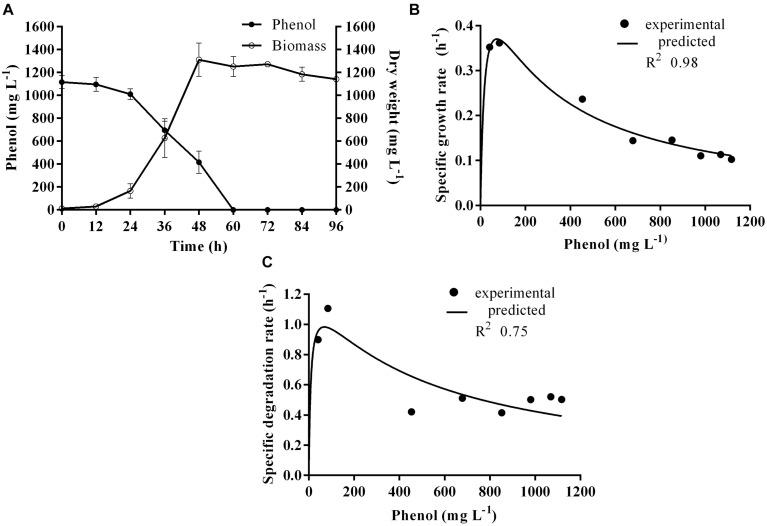
**(A)** Time course of growth and phenol degradation by *G. nicotianae* MSSRFPD35 at initial phenol **(B)** relationship between specific growth rate (μ), and **(C)** specific degradation rate (*q*) and initial substrate concentration (*S*_i_). Haldane’s model simulation was fitted to the experimental values of μ and *q*.

**FIGURE 4:**
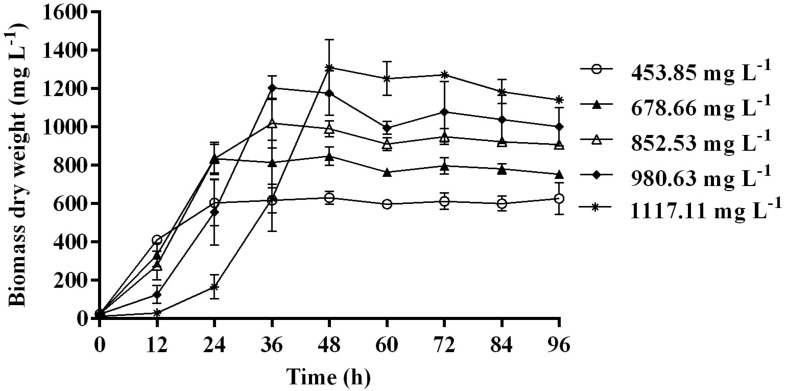
Biomass of MSSRFPD35 at a various phenol substrate concentration.

**FIGURE 5:**
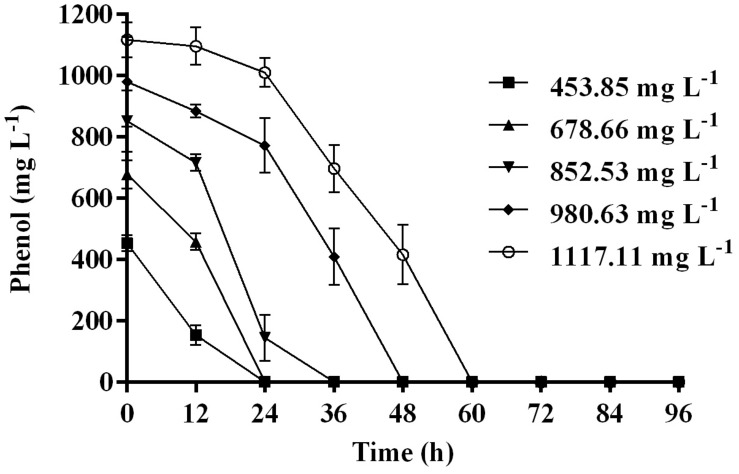
Time course of phenol degradation by MSSRFPD35 at different concentrations of phenol.

Time course assay of *G. nicotianae* MSSRFPD35 revealed that degradation of phenol at conc. range of 41.23 to 1117.11 mg L^–1^ was reached within 6 and 60 h, respectively, with maximum biomass of 1312 mg L^–1^ (Figure 3A). The biomass at different initial phenol concentration measured every 12 h at OD 600 nm was converted into dry biomass (mg L^–1^) to calculate growth rate (Figure 4). Haldane model showed a correlation coefficient *R*^2^ value of 0.98 when inhibiting phenol concentrations were used as substrate and is well fitted with experimental data (Figure 3B). The predicted kinetic parameters were μ^∗^ 0.574 h^–1^, *K*_s_ 20.29 mg L^–1^ and *K*_i_ 268.1 mg L^–1^. The values of kinetic parameters μ^∗^ predicted maximum growth rate, *K*_i_ inhibition constant and *K*_s_ half-saturation constant help in identification of substrate’s inhibition character. True maximum growth rate (μ_max_) and substrate (*S*_m_) concentration at which it occurred were calculated by Eqs. (7) and (6) as 0.37 h^–1^ and 73.76 mg L^–1^, respectively. Here graphically predicted growth rate μ^∗^ (0.574) and true growth rate μ_max_ (0.37) which were overestimated by 55% are shown in Table 1. Depletion of phenol concentration calculated for every 12 h up to 96 h was used to calculate the degradation rate of phenol (Figure 5). Degradation rate for each *S*_i_ was calculated from the slope of the plot between negative logarithmic substrate concentration, −ln(*S*/*S*_i_) and time, *t*. The *q*_s_ values clearly showed an inhibition effect which was reduced when *S*_i_ was increased. The kinetic parameters were *q*^∗^ 1.244, *K*_s_′ 9.152, *K*_i_′ 517.5, *S*_m_′ 68.820 and true maximum degradation rate *q*^*max*^ 0.983 obtained (Table 2) with correlation coefficient value of 0.75 (Figure 3C).

**TABLE 1:** Growth kinetic fitting parameter of MSSRFPD35 and calculated parameters of different microbes grown in phenol.

Bacteria	Parameters obtained for specific growth rate (μ)						References
	μ_max_	*K*_s_	*K*_i_	*S*_m_	μ*_max_	*R*^2^	
*G. nicotianae* MSSRFPD35	0.574	20.29	268.1	73.76	0.37	0.98	This study
*P. putida* LY1	0.217	24.40	121.70	54.50	0.114	0.96	Li et al. (2010)
*P. putida* MTCC 1194	0.109	53.20	148.60	88.90	0.050	0.91	Mathur and Majumder (2010)
*Alcaligenes* sp. TW1	0.58	10.00	550.00	74.20	0.457	–	Essam et al. (2010)
*A. faecalis* B6-2	0.48	469.23	188.16	297.10	0.12	0.90	Heilbuth et al. (2015)
*A. johnsonii* D1	0.55	483.83	2582.63	1117.80	0.29	0.96	Heilbuth et al. (2015)
*B. brevis*	0.078	29.31	2434.70	267.10	0.064	0.95	Arutchelvan et al. (2006)
*S. solfataricus* 98/2	0.094	77.70	319.40	157.50	0.047	0.95	Christen et al. (2012)
*B. cereus* MTCC 9817	0.4396	129.40	637.80	287.28	0.2312	0.81	Banerjee and Ghoshal (2010a)
*Gulosibacter* sp. YZ4	0.601	70.87	418.20	–	–	0.98	Zhai et al. (2012)
*P. variotii* JH6	0.312	130.40	200.00	161.493	0.11931	0.95	Wang et al. (2010)
*C. tropicalis* PHB5	0.3407	15.81	169.00	51.69	0.2113	0.99	Basak et al. (2014)

**TABLE 2:** Degradation kinetic fitting parameter and calculated parameters of different microbes grown in phenol.

Microorganisms	Parameters obtained for specific degradation rate (*q*)						References
	q*	*K*_s_′	*K*_i_′	*S*_m_′	*q*_max_	*R*^2^	
*G. nicotianae* MSSRFPD35	1.244	9.152	517.5	68.820	0.983	0.7542	This study
*B. cereus* MTCC 9817	27.85	59,150	2.411	377.63	0.089	0.64	Banerjee and Ghoshal (2010b)
*B. cereus* MTCC 9818	1.635	9.706	3873.00	193.88	1.486	0.82	Banerjee and Ghoshal (2010b)
*Stenotrophomonas maltophilia* CUPS-3	58.80	1688.00	0.868	38.27	0.659	0.84	Pal et al. (2014)
*Pseudomonas* sp. CUPS-2	34.39	850.50	0.878	27.32	0.544	0.98	Pal et al. (2014)
*P. aeruginosa* CUPS-5	14.31	252.60	2.468	24.96	0.674	0.94	Pal et al. (2014)
*S. solfataricus* 98/2	–	130.30	291.10	174.9	0.110	0.93	Christen et al. (2012)
*C. tropicalis* PHB5	0.2766	2.819	2093.00	76.81	0.257	0.82	Basak et al. (2014)

### Molecular and Metabolic Pathways Involved in Phenol Degradation

The genes coding for catechol 1,2-dioxygenase and catechol 2,3-dioxygenase that are involved in the degradation of phenol in MSSRFPD35 showed amplification of 409 bp and 816 bp, respectively. The BLASTX analysis of the amplified PCR product sequence showed similarity to catechol 1,2-dioxygenase with 98.25% identity to *Glutamicibacter arilaitensis* Re117^*T*^ and *Arthrobacter* sp. W1, followed by 97.3%, to *Arthrobacter* sp. MYb213 and *Glutamicibacter* sp. BW77 (Supplementary Figure S3). The sequence analysis of the amplified product of catechol 2,3-dioxygenase gene showed 96% identity with catechol 2,3-dioxygenase of *G. mysorens*, followed by 96.43% identity with 3,4-dihydroxyphenylacetate 2,3-dioxygenase of *G. arilaitensis* Re117^*T*^ (Supplementary Figure S4). The enzymatic assay of MSSRFPD35 culture extract confirmed that it follows the *ortho* cleavage pathway by increasing absorbance at 260 nm from 0.035 to 0.079 by the formation of aromatic ring cleavage product *cis, cis* muconic acid. Enzyme activity of MSSRFPD35 toward catechol 1,2-dioxygenase calculated was 0.046 μmol min^–1^ and specific activity 0.008 μmol min^–1^ μg^–1^, whereas compounds denoting *meta* cleavage were not detected which was confirmed by no increase in absorbance at 375 nm and no specific enzyme activity was observed. It indicates that isolate MSSRFPD35 follows *ortho* cleavage pathway for the degradation of phenol though the genome information for *meta* pathway was also detected.

### Phenol Degrading Efficiency of MSSRFPD35 in the Presence of Different Heavy Metals

**FIGURE 6:**
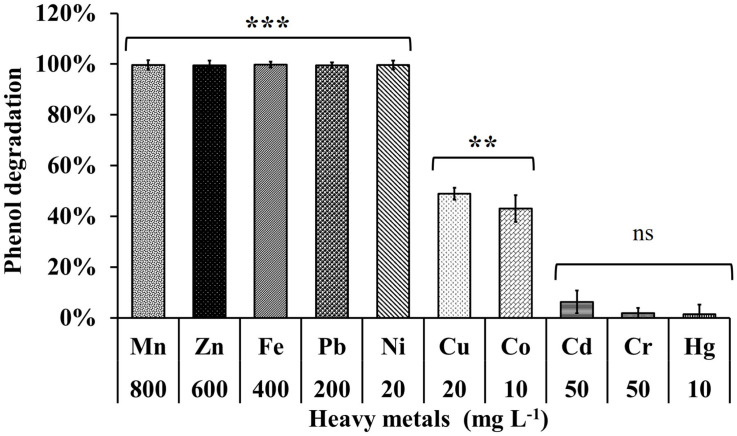
Phenol degradation profiles of MSSRFPD35 under the influence of different heavy metals. Values are mean ± SD of triplicate sets, **P-value = 0.01–0.001, ***P-value = less than 0.001 and ^ns^*P*-value greater than 0.05 represent the significant difference according to Duncan multiple range test compared to control.

The phenol degrading efficiency of MSSRFPD35 was not affected in the presence of heavy metal ions such as Mn (800 mg L^–1^), Zn (600 mg L^–1^), Pb (200 mg L^–1^), Ni (20 mg L^–1^). The phenol degradation was 99% in the presence of heavy metals Pb and Ni, 83% and 93% in the presence of Mn and Zn, respectively, with the time frame of 72 h (Figure 6). Heavy metal Cu (20 mg L^–1^) and Co (10 mg L^–1^) amended medium had an inhibitory effect on phenol degradation, were in only 24% and 43% of phenol were degraded, respectively, in 72 h of incubation. While Cd (50 mg L^–1^), Cr (50 mg L^–1^), and Hg (200 mg L^–1^) completely inhibited the growth of MSSRFPD35 and no phenol degradation was observed. The strain MSSRFPD35 grew in the presence of aromatic compounds like 4-nitrophenol (50 mg L^–1^), gallic acid (200 mg L^–1^), cinnamic acid (100 mg L^–1^), naphthol (50 mg L^–1^), tannic acid (50 mg L^–1^), naphthylamine (50 mg L^–1^), and catechol (100 mg L^–1^) by utilizing all of these as sole carbon source, but was not able to utilize 1-chloro-2,4-dinitrobenzene and failed to grow in the amended medium.

### Soil Microcosm

**FIGURE 7:**
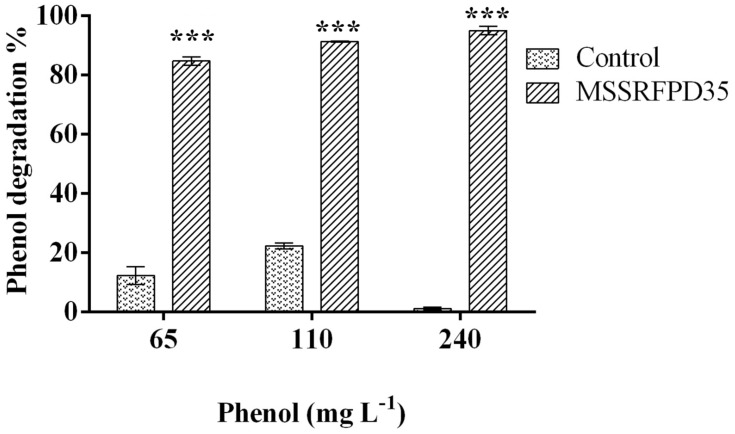
Degradation level of phenol by MSSRFPD35 in sterile soil microcosm. Values are mean ± SD of triplicate sets, ^∗∗∗^*P-*value = less than 0.001 represent the significant difference according to Duncan multiple range test compared to control.

The microcosm experiment conducted with soil slurry spiked with 65 mg L^–1^, 110 mg L^–1^ and 240 mg L^–1^ of phenol showed ∼84% degradation when inoculated with MSSRFPD35 compared to uninoculated soil samples at 10th day of incubation. In the soil slurry amended with 240 mg L^–1^ of phenol showed 94.9% degradation, followed by 84% of degradation of 65 mg L^–1^ and 91% of 110 mg L^–1^ degraded in 10 days of incubation (Figure 7). Control soils without bacterial inoculum showed no significant change in phenol concentration indicating no physiological degradation. Hence, MSSRFPD35 had the potential to degrade phenol not only under laboratory conditions but also in contaminated soils.

## Discussion

Globally, contamination of phenol and its derivatives in soil and water from industrial effluents are increasing which have major toxic effects to all living organisms (Prasse et al., 2018; Wu et al., 2018). Bioremediation of toxic pollutants and inorganic components by microorganisms is a cost-effective and environmentally safe approach (Satchanska et al., 2015; Tiwari et al., 2017). Gu (2016) discussed the key components for efficient biodegradation of environmental pollutants, and hence understanding of the microbial system with the knowledge on metabolism, nutrient utilization, growth rate and subsequently kinetics are essential. This study for the first time attempted to isolate potential phenol degrading bacteria from distillery effluent contaminated *C. indica* rhizospheric soils. Among the microorganisms, bacterial species are often dominantly involved in the degradation of phenol and its derivatives (Whiteley et al., 2001; Banerjee and Ghoshal, 2010a; Pal et al., 2014; Shahryari et al., 2018; Wu et al., 2018). Our study also revealed the presence and abundance of diverse potential phenol degrading *Arthrobacter* strains associated with rhizosphere soils of *C. indica.* The 16S rRNA analysis of all the cluster groups showed similarity to the strain *G. nicotianae*, reclassification of *Arthrobacter nicotianae* (Busse, 2016) but the BOX-DNA fingerprinting analysis significantly showed the existence of an intra-species population. Ellegaard and Engel (2016) reported that intra-species variation cannot be resolved using 16S rRNA based analysis because of high sequence conservation in the 16S rRNA gene. The strains showed different degrees of phenol degradation indicating the intra-species genetic variation in the BOX PCR fingerprinting pattern. The genetic variation among the clustered identical isolates might exhibit significant variation in the phenol degradation efficiency. This indicates that the genotypic and phenotypic plasticity of the strains is due to the lateral gene transfer which was observed in several other genera associated in different niches (Whiteley et al., 2001; Bedhomme et al., 2019).

Among the bacterial genera, *Arthrobacter* sp. class 1 microorganism are reported as key candidates involved in the degradation of phenolic compounds habited in soil and rhizosphere regions (Arora and Sharma, 2015; TRBA, 2015; Wang et al., 2015; Busse, 2016). Studies using *Arthrobacter nicotianae* sp. W1 which preferentially grew on ethylbenzene, toluene, catechol, benzene, xylene, and cresol involved a synergistic mechanism in the degradation process (Ma et al., 2013; Wong et al., 2015) of pentachloronitrobenzene (Wang et al., 2015). In this study, we are reporting *G. nicotianae* MSSRFPD35 with potential to degrade phenol up to 1113 mg L^–1^ within 60 h under minimal nutrient conditions, comparatively this is one of the efficient phenol degrading strain reported so far. Among the *G. nicotianae* isolates from this study, the degree of phenol degradation varied significantly, which indicates that degradation potential is not strongly associated with the genus (or) species. The potential of phenol degradation is a specific trait of the individual strain. Several studies reported that degree of phenol degradation and other functional traits varies among the same group, due to its habitat, presence of respective gene and its level of expression (Wang et al., 2015; Tian et al., 2017; Bedhomme et al., 2019). Strain *Arthrobacter citreus* was reported to degrade 470 mg L^–1^ phenol in 24 h (Karigar et al., 2006); *Arthrobacter chlorophenolicus* A6 has potential to degrade high concentrations of 4-CP (up to 347 mg L^–1^), 4-nitrophenol and 4-bromophenol phenol derivatives (Westerberg et al., 2000). Phenol degradation by *Acinetobacter* sp. SA01 isolated from farmland contaminated with pesticides and oil refinery pollutants was observed at 1000 mg L^–1^ of phenol after 60 h under the optimum condition of pH 7, 30°C and 180 rpm (Shahryari et al., 2018). *P. putida* MTCC 1194 degrade phenol at concentration of 1000 mg L^–1^ in 162 h (Kumar et al., 2005). Strain *Kocuria* sp. TIBETAN4 isolated from hyper-saline and alkaline soda lake soil could degrade 470.5 mg L^–1^ phenol within 3 days, 705 mg L^–1^ phenol within 4 days, but the degradation of 941 mg L^–1^ phenol was lengthened to 10 days for complete degradation (Wu et al., 2018).

In the time course assay lag phase of *G. nicotianae* MSSRFPD35 extended with increasing phenol concentration in the medium, which prolonged the time of biodegradation. Shahryari et al. (2018) reported 18 h lag phase stage of *Acinetobacter* sp. SA01 and *Pseudomonas* sp. NCCP-407 to degrade 1000 mg L^–1^ and 750 mg L^–1^ of phenol, respectively. Christen et al. (2012) stated that the bacterial cultures that were acclimatized in appropriate substrate concentration had less or no lag phase. However, MSSRFPD35 had no lag phase up to 980 mg L^–1^ of phenol, but increasing concentration up to 1117 mg L^–1^ of phenol had 12 h of lag phase which depict the efficiency of MSSRFPD35 to acclimatize to high phenol concentrations. Increasing concentration of phenol can influence changes in the cell membrane and protect the cell from toxic effects of phenol (Murinova and Dercova, 2014) also the cell takes a longer time to adapt to phenol toxicity. The specific growth rate of MSSRFPD35 strain was higher than *P. putida* LY1 (Li et al., 2010), *Bacillus brevis* (Arutchelvan et al., 2006) and *Sulfolobus solfataricus* 98/2 (Christen et al., 2012). It was almost equivalent to earlier reported potential phenol degrading strains like *Alcaligenes* sp. TW1 (Essam et al., 2010) and *B. cereus* MTCC 9817 (Banerjee and Ghoshal, 2010b). *Gulosibacter* sp. YZ4 has a higher specific growth rate of 0.6 mg L^–1^ which can degrade 2000 mg L^–1^ of phenol within 72 h (Zhai et al., 2012). Similarly, MSSRFPD35 can degrade 1100 mg L^–1^ of phenol within 60 h.

The *K*_s_ values which depict the affinity toward the phenol substrate was on the lower side for MSSRFPD35 and is equivalent to *P. putida* LY1, *Candida tropicalis* PHB5 and *Bacillus brevis*. Inhibition constant *K*_i_ for *G. nicotianae* MSSRFPD35 was in medium-range among reported strains and it is similar to *Paecilomyces variotii* JH6 (Wang et al., 2010) and *S. solfataricus* 98/2 (Christen et al., 2012). The *K*_i_ value in this study indicates the higher tolerance of MSSRFPD35 toward phenol when compared to other bacteria reported. Higher values of *K*_i_ denote lower inhibition which leads to Monod kinetic model and also indicates the concentration up to which the bacterial strain can tolerate shock loads (Bajaj et al., 2009). Inconsistency among the predicted (μ^∗^) and true (μ_max_) maximum growth rate were demonstrated in earlier studies conducted using substrate phenol (Christen et al., 2012; Basak et al., 2014). In this study, graphically predicted μ^∗^ (0.574) and true growth rate μ_max_ (0.37) were overestimated. However, compared to other bacteria the μ_max_ of MSSRFPD35 was second higher after *Alcaligenes* sp. TW1 (Essam et al., 2010). Though *S*_m_ of MSSRFPD35 was lower among few previous studies, it was higher than *C. tropicalis* PHB5 and *P. putida* LY1 but equal to *Alcaligenes* sp. TW1. This indicates that phenol concentration of 73.76 mg L^–1^ is the optimal substrate concentration for bacterial growth, while increment in substrate concentration above this inhibited the bacterial growth and biomass formation. Higher values of specific growth rate denoted that MSSRFPD35 can utilize the phenol in a comparatively lesser duration of 60 h. Though specific degradation rate is usually independent of *S*_i_, phenol as a substrate showed inhibitory effect on specific degradation rate *q*_s_ in different studies (Yan et al., 2005; Bai et al., 2007).

Molecular analysis showed the MSSRFPD35 catechol 1,2-dioxygenase gene sequence confirms the similarity to *Glutamicibacter* spp., while catechol 2,3-dioxygenase enzyme-coding gene sequence showed similarity only with catechol 2,3-dioxygenase of *G. mysorens.* The sequence results did match with 3,4-dihydroxyphenylacetate and 2,3-dioxygenase gene of *Glutamicibacter* sp. Even though primers were designed to target a highly specific region of catechol 2,3-dioxygenase enzyme-coding gene, it was not depicted in blast analysis. Its due to the diversity in the enzyme gene sequence regions which is highly variable with less similarity to the target enzyme sequence, whereas both enzymes, homoprotocatechuate 2,3-dioxygenase and catechol 2,3-dioxygenase belongs to the same family of oxidoreductases and is involved in decyclizing of aromatic ring (Sandhu et al., 2009; Tian et al., 2017). The results confirmed that MSSRFPD35 and other *Glutamicibacter* sp. in this study might possess coding regions for both catechol 1,2-dioxygenase and catechol 2,3-dioxygenase enzymes. Though the gene coding for catechol 2,3-dioxygenase was amplified from the genomic DNA of MSSRFPD35, only catechol 1,2-dioxygenase enzymatic activity was detected indicating that MSSRFPD35 adopted *ortho* pathway to breakdown catechol intermediate and not *meta* pathway. Similarly in *S. solfataricus* 98/2 phenol degradation adopted the meta pathway with catechol 2,3-dioxygenase enzyme, though it possesses both catechol 1,2-dioxygenase and catechol 2,3-dioxygenase genes (Comte et al., 2013). Alkylphenol degrading *Pseudomonas* sp. TX1, and *P. putida* TX2 were reported to possess coding genes for both the enzymes (Tuan et al., 2011).

*Glutamicibacter* spp. were reported to degrade aromatic derivatives like pentachlorobenzene, *p*-cresol, benzene and toluene, even though the toxicity of aromatic compounds depends on other functional groups attached to the derivatives, most of them formed intermediate in the form of catechol derivatives (Vikram et al., 2013). Wang et al. (2009) reported *G. nicotianae* W1 adopted *ortho* pathway in the degradation of phenol *p*-cresol and mixed phenolic compounds. Presence of catechol dioxygenase enzyme in MSSRFPD35 evidenced that the catechol derivatives could be cleaved and can be converted to simpler forms. For example, distillery effluent contains phenolic acids and its derivatives like gallic acid, cinnamic acids and also other aromatic derivatives that are abundantly used in pesticide, plastic, explosives petrochemical and organic synthesis industries (Arora and Jain, 2012).

The strain MSSRFPD35 could degrade high levels of synthetic phenol in the presence of different heavy metals; present in industrial effluents that are predominantly contaminated with phenolic and other aromatic derivatives. It is an added functional property of the strain and the application may be expanded in the bioremediation of effluents discharged from the industries. Phenol degrading efficiency in the presence of heavy metals is an important criteria for a strain as these heavy metals are released as co-contaminates along with phenol that would inhibit the bacterial growth and decrease the biodegradation efficiency (Thavamani et al., 2012; Wong et al., 2015). Majority of the heavy metals are reported to be toxic to many bacterial groups by altering the cell morphology, disrupting the cell membrane, directly inhibiting the electron transport enzyme activity, decreasing the biomass, inhibiting growth and damaging the nucleic acid structure (Nies, 1999; Bruins et al., 2000; Sandrin and Maier, 2003; Janicka-Russak et al., 2008). But certain bacterial groups exhibit tolerance to multiple heavy metals at varied concentrations. The major mechanism involved in tolerance is through intracellular and extracellular metal sequestration, metal oxidation, methylation, demethylation, metal-organic complexion, metal-ligand degradation, exclusion by permeability barrier, and production of metal chelators like metallothioneins and exopolysaccharide (Ramasamy and Banu, 2007; Igiri et al., 2018). Silva et al. (2012) reported that the presence of heavy metals Fe^3+^ and Mn^2+^ stimulated the catechol dioxygenase activity of *Gordonia polyisoprenivorans.* Copper metal has a negative reaction toward Fe-S enzymes like ring-hydroxylating dioxygenases and intradiol cleavage dioxygenase which are involved in aromatic metabolic pathway (Grass et al., 2011). Yeom and Yoo (1997) reported that benzene and toluene degradation by *Alcaligenes xylosoxidans* Y234 were highly inhibited by Co^2+^ and Ag^+^ and Cu^2+^ which affects the catechol 1,2 dioxygenase enzymatic reaction. Lima et al. (2008) reported that *Burkholderia cepacia* utilized phenol (100 μg ml^–1^) in the presence of K_2_Cr_2_O_7_ at the concentration of 100 to 200 μg ml^–1^. Similarly, El-Naas et al. (2009) reported biodegradation of phenol by *P. putida* at the concentration of 150 mg L^–1^ and reported heavy metal ions such as iron, aluminum and zinc had no effect on the phenol biodegradation rate. Ontanon et al. (2015) reported degradation of phenol by *Bacillus* at maximum concentrations of 1000 mg L^–1^ and reduced Cr (VI) to Cr (III). This study revealed the potential of MSSRFPD35 in degrading phenol in the presence of different heavy metals. Satchanska et al. (2015) also reported that bacterial strains *B. subtilis* KCMRG5 and *P*. *rhodesiae* KCMR5 were able to degrade phenol in the presence of heavy metals. *P. fluorescens* (BBN1) and *P. corrugata* (BBB2) were reported to degrade PAH which were able to tolerate 993.6 mg L^–1^-Pb (NO_3_)_2_, 717.83 mg L^–1^-ZnSO_4_ and 499.36 mg L^–1^-CuSO_4_, respectively (Máthé et al., 2012). However, no evaluation of heavy metal effect on phenol biodegradation by *G. nicotianae* has previously been performed. The present study indicates that *G. nicotianae* MSSRFPD35 strain can degrade phenol in the presence of Pb, Ni, Cu, Co, Mn, and Zn metals which might occur as co-pollutants in industrial effluent. Soil microcosm studies also confirmed the *in vivo* bioremediation potential of MSSRFPD35 and to degrade phenol in contaminated soils. Though there are no studies on soil microcosm with phenol as a substrate, some studies reported 90% degradation of chloro-nitro phenol by *Burkholderia* sp. RKJ 800 (Arora and Jain, 2012) and *Cupriavidus* sp. a3 (Tiwari et al., 2017) and 2 nitrobenzene by *Arthrobacter* sp. SPG in soil (Arora and Sharma, 2015). Strain MSSRFPD35 holding a capacity of tolerating heavy metals, degrading relatively high concentrations of phenol in a shorter time and other phenol derivatives showed promising prospect for application in the remediation of phenol contaminated sites.

## Conclusion

The strain *G. nicotianae* MSSRFPD35 proved to degrade phenol at relatively high concentration via catechol 1,2-dioxygenase directed *ortho* pathway, specifically in the presence of different heavy metals. The growth and degradation kinetics of the strain MSSRFPD35 utilizing phenol as a sole carbon and nutrient source was well characterized by Haldane model where the μ_max,_ *K*_i_ and *K*_s_ were described. The strain was capable of growing in various monocyclic and polycyclic aromatic hydrocarbons, tolerate and degrade phenol in the presence of heavy metals like lead, zinc, manganese, iron, and nickel. The versatility of this strain can be exploited for promoting it as a renewable resource for biodegradation of phenol and its derivatives co-contaminated with heavy metals discharged from various industries.

## Data Availability Statement

The datasets presented in this study can be found in online repositories. The names of the repository/repositories and accession number(s) can be found in the article/Supplementary Material.

## Author Contributions

PD collected the samples, designed and performed the experiments, data analysis and drafted the manuscript. JS and AA supported sample collection, contributed to *in vitro* experiments, molecular analysis and manuscript correction. PR supervised and supported the study, revised the manuscript and approved for publishing. All authors contributed to the article and approved the submitted version.

## Conflict of Interest

The authors declare that the research was conducted in the absence of any commercial or financial relationships that could be construed as a potential conflict of interest.
