# Supplementary material for: Kinetics of Phenol Biodegradation by Heavy Metal Tolerant Rhizobacteria Glutamicibacter nicotianae MSSRFPD35 From Distillery Effluent Contaminated Soils
Source: Front Microbiol. 2020 Jul 15;11:1573. doi: 10.3389/fmicb.2020.01573 (PMC7373764; doi:10.3389/fmicb.2020.01573)
Supplement: Supplementary file 1 [file Data_Sheet_1.docx]

**Kinetics of phenol biodegradation by heavy metal tolerant rhizobacteria *Glutamicibacter nicotianae* MSSRFPD35 from distillery effluent contaminated soils**

Purushothaman Duraisamy, Jegan Sekar, Anu Dorothy Arunkumar, Prabavathy Vaiyapuri Ramalingam*

Microbiology lab, M.S. Swaminathan Research Foundation, 3^rd^ cross road, Taramani institutional area, Taramani, Chennai-600 113, India.

***Corresponding author:**

Dr. Prabavathy Vaiyapuri Ramalingam

E-mail: [prabavathyvr@mssrf.res.in](mailto:prabavathyvr@mssrf.res.in)

List of figures

Fig S1. Standard linear regression equation at different concentrations of phenol

**
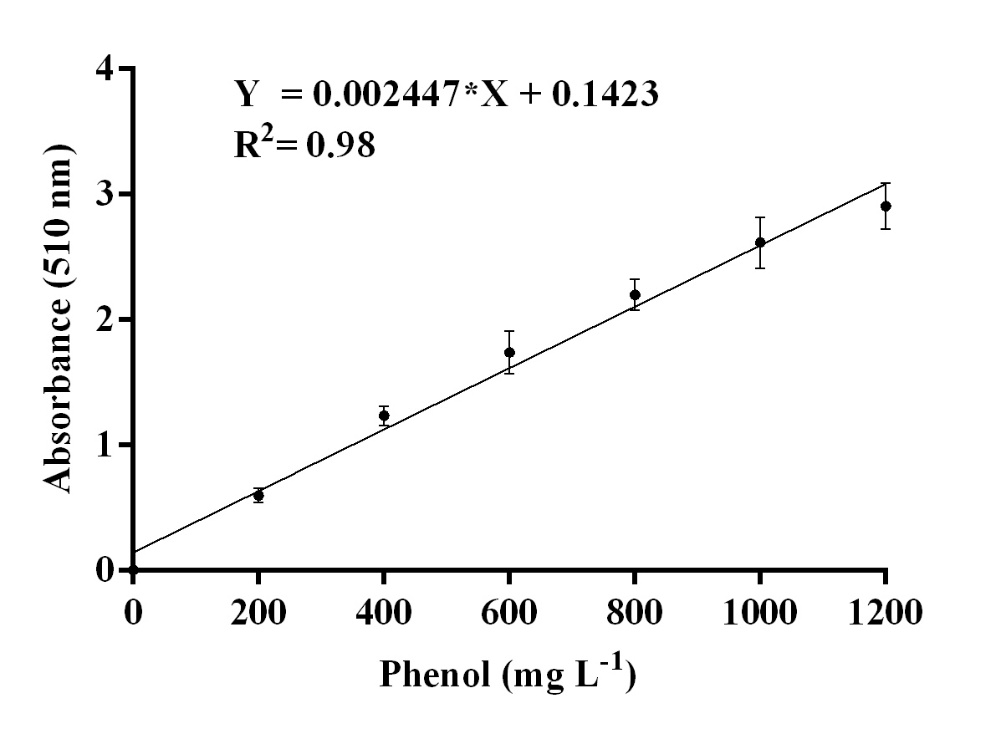
**

Fig S2. BOX-PCR generated dendrogram of phenol degrading bacterial isolates. Dendrogram showing the genetic relatedness between the isolates determined by analysis of BOX-PCR fingerprint patterns using the Jaccard similarity coefficient and UPGMA algorithm.


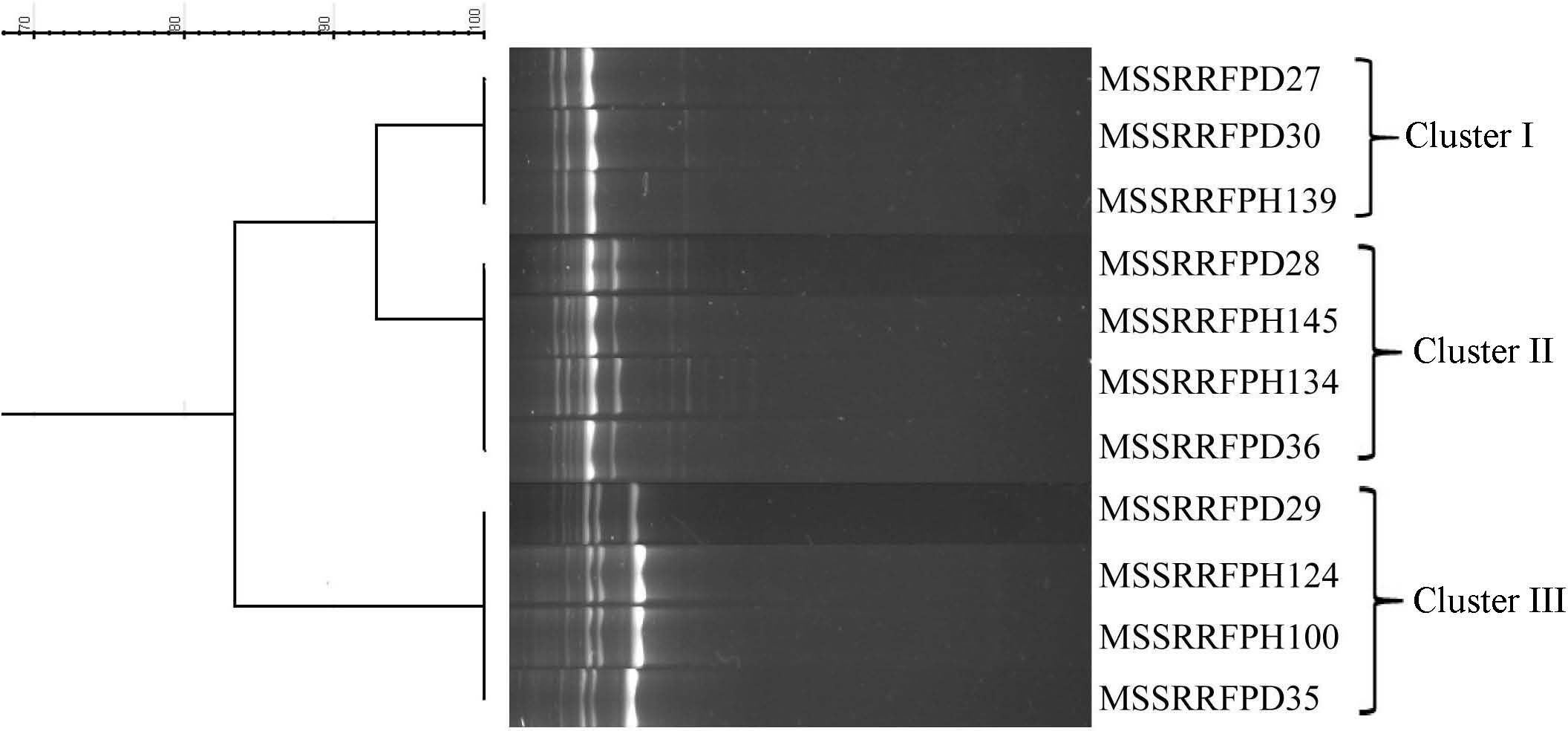


Fig. S3. Neighbour-joining tree based on partial catechol 1,2-dioxygenase gene sequences of strains MSSRFPD35 with closely related catechol 1,2-dioxygenase and chlorocatechol 1,2-dioxygenase sequences from related organisms.

**
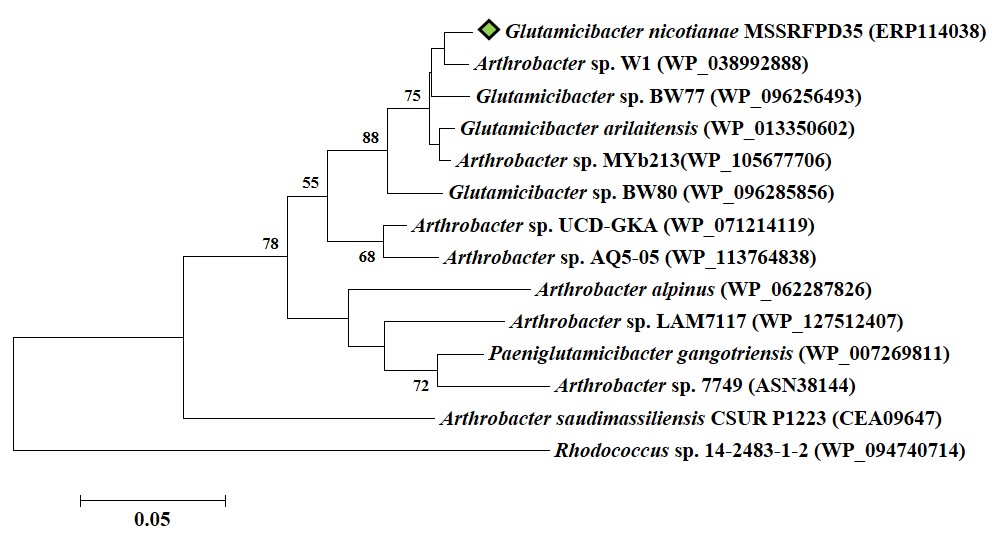
**

Fig. S4. Neighbour-joining tree based on partial catechol 2,3-dioxygenase gene sequences of strains MSSRFPD35 with closely related catechol 2,3-dioxygenase and 3,4-dihydroxyphenylacetate 2,3-dioxygenase sequences from related organisms.

**
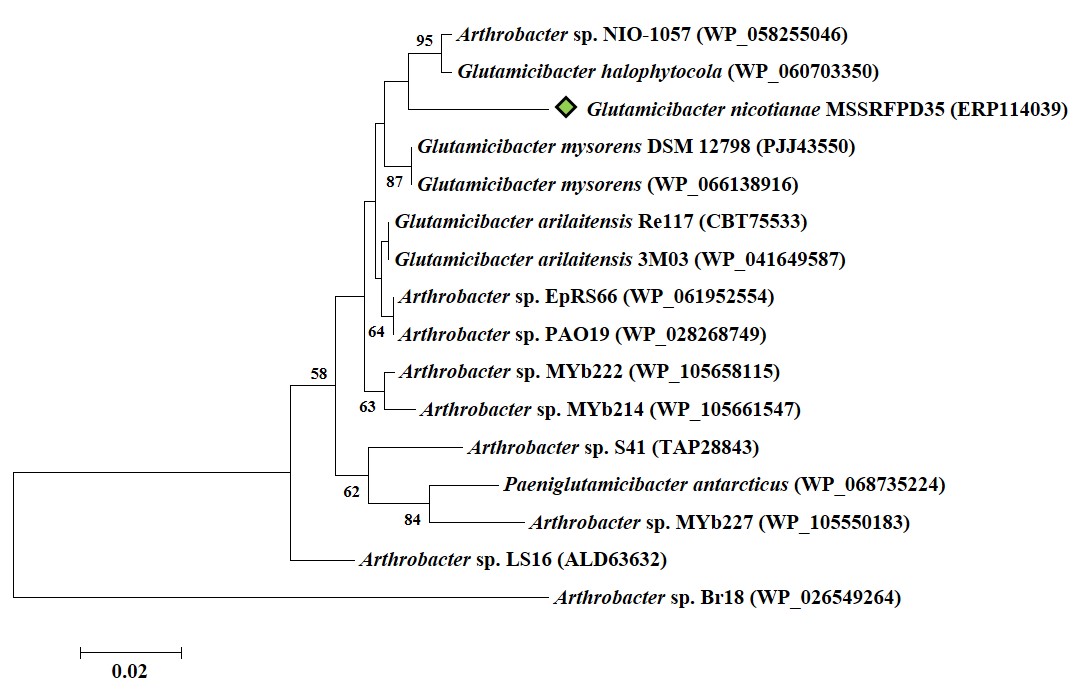
**
